# A Reference Genome Assembly of American Bison, *Bison bison bison*

**DOI:** 10.1093/jhered/esab003

**Published:** 2021-02-17

**Authors:** Jonas Oppenheimer, Benjamin D Rosen, Michael P Heaton, Brian L Vander Ley, Wade R Shafer, Fred T Schuetze, Brad Stroud, Larry A Kuehn, Jennifer C McClure, Jennifer P Barfield, Harvey D Blackburn, Theodore S Kalbfleisch, Derek M Bickhart, Kimberly M Davenport, Kristen L Kuhn, Richard E Green, Beth Shapiro, Timothy P L Smith

**Affiliations:** 1 Department of Biomolecular Engineering, University of California Santa Cruz, Santa Cruz, CA, USA; 2 USDA, ARS, Animal Genomics and Improvement Laboratory, Beltsville, MD, USA; 3 USDA, ARS, U.S. Meat Animal Research Center, Clay Center, NE, USA; 4 Great Plains Veterinary Educational Center, University of Nebraska–Lincoln, Lincoln, NE, USA; 5 American Simmental Association, Bozeman, MT, USA; 6 Simmentals of Texas, Granbury, TE, USA; 7 Stroud Veterinary Embryo Services, Weatherford, TE, USA; 8 USDA, ARS, U.S. Dairy Forage Research Center, Madison, WI, USA; 9 College of Veterinary Medicine and Biomedical Sciences, Colorado State University, Fort Collins, CO, USA; 10 USDA, ARS, National Animal Germplasm Program, Fort Collins, CO, USA; 11 Gluck Equine Research Center, University of Kentucky, Lexington, KY, USA; 12 Department of Animal, Veterinary, and Food Science, University of Idaho, Moscow, ID, USA; 13 Department of Ecology and Evolutionary Biology, University of California Santa Cruz, Santa Cruz, CA, USA; 14 Howard Hughes Medical Institute, University of California Santa Cruz, Santa Cruz, CA, USA

**Keywords:** bovine, interspecies hybrid, nanopore sequencing, trio binning, Genome resources

## Abstract

Bison are an icon of the American West and an ecologically, commercially, and culturally important species. Despite numbering in the hundreds of thousands today, conservation concerns remain for the species, including the impact on genetic diversity of a severe bottleneck around the turn of the 20th century and genetic introgression from domestic cattle. Genetic diversity and admixture are best evaluated at genome-wide scale, for which a high-quality reference is necessary. Here, we use trio binning of long reads from a bison–Simmental cattle (*Bos taurus taurus*) male F_1_ hybrid to sequence and assemble the genome of the American plains bison (*Bison bison bison*). The male haplotype genome is chromosome-scale, with a total length of 2.65 Gb across 775 scaffolds (839 contigs) and a scaffold N50 of 87.8 Mb. Our bison genome is ~13× more contiguous overall and ~3400× more contiguous at the contig level than the current bison reference genome. The bison genome sequence presented here (ARS-UCSC_bison1.0) will enable new research into the evolutionary history of this iconic megafauna species and provide a new tool for the management of bison populations in federal and commercial herds.

The American plains bison (*Bison bison bison*), an iconic symbol of the American West, is of significant evolutionary (Guthrie 1970), ecological (Hartnett et al. 1996), commercial (Yorks and Capels 1998), and cultural (Torbit and LaRose 2001) interest, as well as of conservation concern (Freese et al. 2007). First appearing in the early Pleistocene in Eurasia (Massilani et al. 2016), steppe bison (*Bison priscus*) are thought to be the ancestors of both extant species of *Bison*, the American bison (*B. bison*) and European bison (*B. bonasus*) (Guthrie 1970; Gautier et al. 2016; Wang et al. 2018). Paleoecological, geochronological, and molecular dating using mitochondrial genomes suggest steppe bison first arrived in North America ~200–150 thousand years ago (Froese et al. 2017). Following their arrival, they expanded throughout the continent and underwent a period of extensive diversification, represented by the appearance of a number of distinct morphologies. Bison populations declined prior to the Last Glacial Maximum ~20 thousand years ago, eventually becoming extinct across the northern part of their range (Shapiro et al. 2004; Heintzman et al. 2016). American bison today all descend from a lineage that persisted south of the Laurentide and Cordilleran ice sheets during this period (Heintzman et al. 2016).

By the second half of the 19th century, bison populations had rebounded from their early Holocene near-extinction and numbered in the millions across the Great Plains (Freese et al. 2007). However, sport hunters and ranchers seeking to establish land suitable for grazing cattle nearly hunted bison to extinction (Hedrick 2009). At the turn of the 20th century, as few as 100 bison remained, and cattle ranchers were attempting to improve hardiness of their stock through hybridization with bison (Boyd 1908; Goodnight 1914). These efforts were mostly unsuccessful, but it is possible that all bison today derive some portion of their ancestry from domestic cattle (Halbert and Derr 2007). Today’s bison are closely managed, largely in commercial herds (Sanderson et al. 2008) but with tens of thousands of individuals in herds designated for conservation (Plumb and Sucec 2006). The impacts on bison genetic diversity of the recent bottleneck, history of admixture, and current management scheme are largely unknown (Hartway et al. 2020).

Despite being well-studied using genetic tools (Ward et al. 1999; Halbert and Derr 2007, 2008; Cronin et al. 2013; Cherry et al. 2019), there currently exists only a highly fragmented bison reference genome (Bison_UMD1.0; GCF_000754665.1). As a consequence, DNA-based study of bison has largely focused on coarse molecular tools such as mitochondria and microsatellites (Ward et al. 1999; Halbert and Derr 2007; Cherry et al. 2019). A high-quality bison genome will provide greater sensitivity for examining genetic diversity among bison herds, as well as enable identification of admixed or reduced diversity regions in bison genomes and investigation of the functional consequences of these regions. This will aid in the study of bison evolutionary history and ecological impact and in the effective management of bison populations for conservation and commercial production.

We present a reference genome of the American plains bison (*B. bison bison*), ARS-UCSC_bison1.0, obtained through trio binning of long reads from a male F_1_ bison–Simmental cattle hybrid fetus. Despite the rapidly increasing quality of reference genomes due to improvements in sequencing technology (Jain et al. 2018; Wenger et al. 2019) and algorithmic advances (Koren et al. 2017; Ruan and Li 2020), genome assemblies still often suffer from errors created when collapsing heterozygous regions of the genome into a single linear sequence, particularly in areas of complex allelic variation (Rhie, McCarthy et al. 2020). Trio binning uses heterozygosity as a strength, rather than weakness, in the assembly process by harnessing parent-specific sequences to sort long reads from an offspring to either parental haplotype (Koren et al. 2018). Assemblies can then be conducted on each parental haplotype separately, avoiding the need to collapse distinct haplotypes into a single sequence. This process results in 2 separate, phased genomes from a diploid individual.

Trio binning is particularly well-suited to assembling genomes from the F_1_ hybrids of interspecific crosses. This is because sorting reads is simplified with increasing evolutionary distance between the 2 parental haplotypes, as it relies on identifying unique sequences within either haplotype. Trio binning also has the advantage that 2 reference genomes are created from sequencing 1 individual (see also Heaton et al. 2021). However, a limitation of this approach is that only 1 of 2 sex chromosomes is assembled for each species. Trio binning of interspecies or inter-subspecies crosses has been used recently to assemble some of the most complete and contiguous vertebrate genomes, including from an Angus/Brahman cattle cross (Koren et al. 2018) and a Highlander cattle/yak cross (Rice et al. 2020). The male haplotype genome sequence we present here is chromosome-scale, highly complete, and as contiguous as the best livestock and model organism reference genomes available.

## Methods

### Biological Materials

#### Ethics Statement

All cattle protocols were approved by the Institutional Animal Care and Use Committee (IACUC) of the University of Nebraska–Lincoln, an AAALAC International Accredited institution (IACUC Project ID 1697). Bison semen collections were approved by the IACUC at Colorado State University, IACUC protocol 17-7117A.

### Animals, In Vitro Fertilization, and Tissue Collection

Semen from a Yellowstone bison bull (tag number 709, SAMN16823422) was collected and a 4-year-old fullblood Simmental female (BHR Lady Sieg C235E, American Simmental Association registration 3182916, SAMN16825967) was selected as the donor based on her representation of the breed and availability as a donor. Five ova from the donor female were aspirated on 16 January 2019 and fertilized in vitro a day later with semen from Yellowstone bison 709. The same day, 5 Simmental heifers were selected as embryo recipients and embryos were implanted on 24 January 2019 and recipients were observed daily for repeat estrus cycling. Recipients were examined with ultrasonography at 28, 54, 75, and 105 days posttransplantation and controlled intravaginal drug release (CIDR) devices containing progesterone were replaced in the pregnant recipients at each event to help maintain pregnancies. Three pregnancies were confirmed at 22 and 54 days, 2 at 75 days, and 1 at 105 days posttransplantation. On 23 May 2019, the male F_1_ fetus was collected by cesarean at 119 days posttransplantation. Lung tissue was flash-frozen in liquid nitrogen and stored at −80 °C until DNA isolation and sequencing.

### Nucleic Acid Library Preparation and Sequencing

DNA was extracted from 50 mg of the F_1_ hybrid fetus frozen lung tissue and long-read sequencing templates were prepared using the Ligation Sequencing Kit LSK-109 (Oxford Nanopore, Oxford, United Kingdom). Seven libraries were sequenced on an Oxford Nanopore PromethION platform across 16 R9.4.1 flow cells. Additional template for ultra-long sequencing was also constructed using a similar approach as above with the LSK-109 kit, with modifications to the DNA handling and cleanup procedure (https://community.nanoporetech.com/posts/rocky-mountain-adventures), and sequenced on 22 Min106 R9.4.1 flow cells with the GridION x5 platform. Raw nanopore signal fast5 files were converted to fastq format using the Guppy v3.5.1 basecaller (available from Oxford Nanopore Technologies via their community site, https://community.nanoporetech.com). For detailed nanopore sequencing methods, see Heaton et al. (2021).

We also constructed Illumina sequencing libraries for each member of the trio. As input material for the libraries, we used the same F_1_ hybrid lung DNA extract, as well as DNA extracted from a semen sample from the bison sire and a blood sample from the Simmental dam. Libraries were constructed using the Tru-Seq PCR-Free Kit (Illumina Inc., San Diego, CA) and sequenced on an Illumina NextSeq500 instrument using a 2 × 150-cycle paired-end kit.

To gather information about genome spatial organization for use in scaffolding, we generated Hi-C libraries by cross-linking approximately 50 mg of fetal lung tissue, performing proximity ligation and capture, and preparing Illumina libraries using the ProximoHi-C v1.5 kit (Phase Genomics, Seattle, WA), following the manufacturer’s recommendations. These libraries were also sequenced on a NextSeq500, with 2 × 150 cycles. Mapping distance and quality statistics of Hi-C read pairs were generated using the program hic_qc (https://github.com/phasegenomics/hic_qc). See Supplementary Table S1 for sequencing data summary.

### Genome Assembly and Annotation

#### Heterozygosity Estimation

To assess heterozygosity of each of the bison sire, Simmental dam, and F_1_ hybrid, jellyfish v1.1.11 (Marçais and Kingsford 2011) was used to count short subsequences (*k*-mers; *k* = 21) present within the shotgun Illumina reads for the parents and Illumina reads for the hybrid. GenomeScope v1.0 (Vurture et al. 2017) was then used to estimate heterozygosity.

### Assembly

For Illumina data from each parent, reads below 75 bp were discarded and low-quality ends of the reads were trimmed using Trimmomatic v0.38 (Bolger et al. 2014) in paired-end mode (LEADING:10 TRAILING:10 SLIDINGWINDOW:4:18 MINLEN:75).

**Figure 1.**
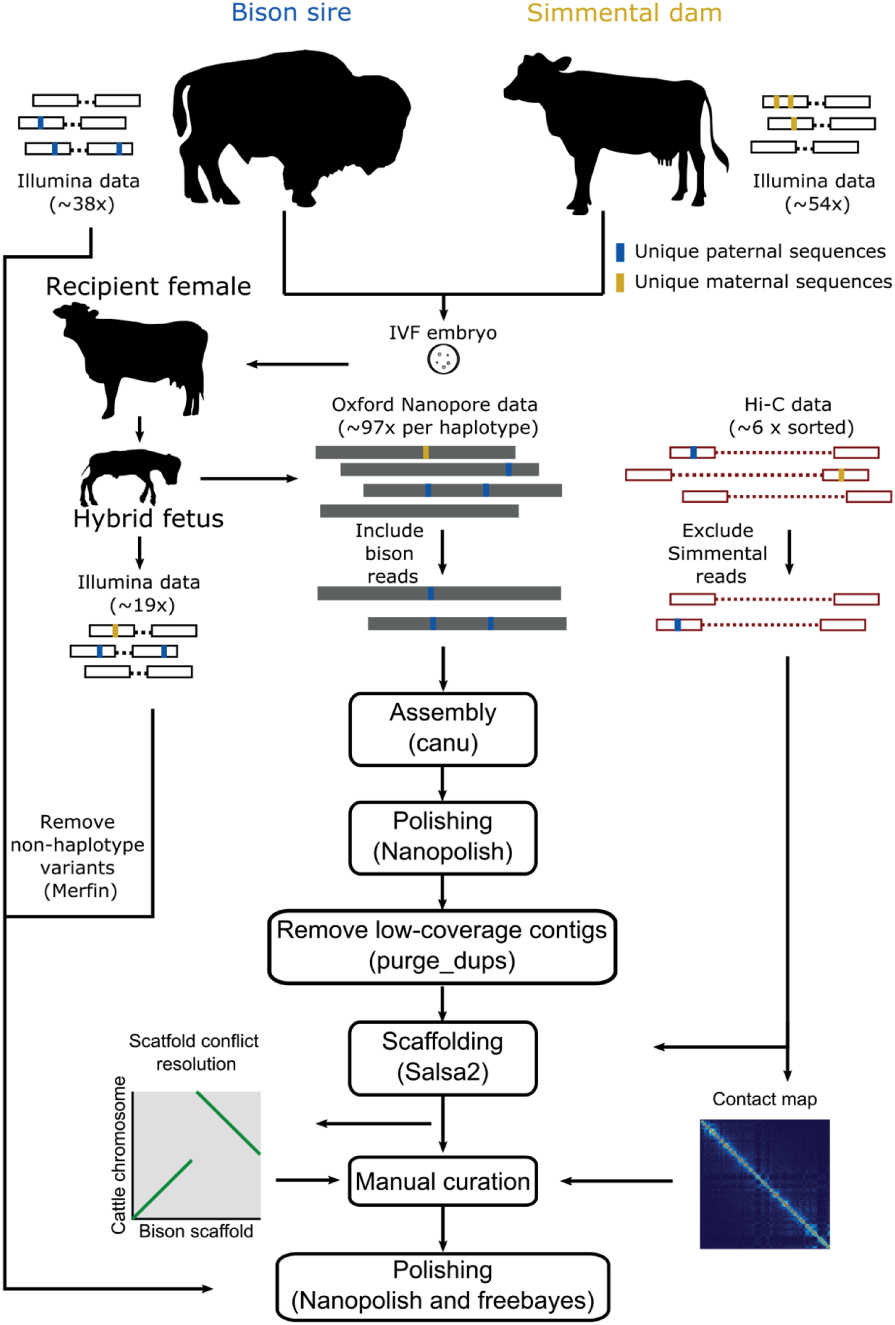
Schematic showing trio binning and assembly process.

An initial assembly was then generated using the trio binning feature implemented in Canu v1.8 (Koren et al. 2017) (see Figure 1 for overview of assembly process; see Table 1 for programs used throughout assembly). First, all *k*-mers (*k* = 21) within parental Illumina reads were counted separately for each parent using meryl v1.0 (Rhie, Walenz et al. 2020). We then sorted the nanopore reads from the F_1_ hybrid using unique *k-*mers present in parental short-read data (see Supplementary File S1 for commands). Those that originated from the bison paternal haplotype were then identified based on unique *k*-mers present a minimum of 6 times in the bison sire short-read data.

**Table 1.** List of programs used for the assembly

Assembly	Program	Version
*K*-mer counting	jellyfish	1.1.11
Heterozygosity estimation	GenomeScope	1
Read trimming	Trimmomatic	0.38
*K*-mer counting	meryl	1
Read binning, error correction, read trimming	Canu	1.8
Unitigging	Canu	1.9
Scaffolding and polishing		
Contig polishing	Nanopolish	0.11.1
Remove low-coverage, duplicated contigs	purge_dups	1.0.1
Long read, genome–genome alignment	minimap2	2.16
Aligning short reads to genome	bwa	0.7.17
Scaffolding	Salsa	2.2
Visualizing genome–genome alignment	D-Genies	1.2.0
SAM/BAM file manipulation	samtools	1.9
Estimate Hi-C library quality	hi_qc	Downloaded 29 June 2019
Generate Hi-C contact matrix	PretextMap	0.1
Visualize Hi-C contact matrix	PretextView	0.01
Fasta manipulation	CombineFasta	0.0.16
Variant calling	freebayes	1.3.1-1-g5eb71a3-dirty
Evaluation		
*K*-mer-based assembly evaluation	Merqury	1
Identify conserved orthologs	BUSCO	v4
*K*-mer-based variant filtration	Merfin	Downloaded October 2020
Read mapping statistics	Lumpy-sv	0.3.0
Alignment feature response curve	FRC_align	1.0.0
VCF/BCF file manipulation	bcftools	1.9
Variant calling	paftools.js	(minimap2 v2.16)
Annotation		
Genome annotation liftover	Liftoff	1.5.1

Initial contigs for the bison genome were assembled using reads assigned to the bison sire haplotype as input for Canu v1.8 that performed the read correction and trimming steps. We switched to Canu v1.9 for the unitigging step, as the latter version corrected an error in the consensus generation process. Contigs were polished using Nanopolish v0.11.1 (Loman et al. 2015), which relies on raw signal data from nanopore reads to derive a more accurate consensus sequence and has been used to generate accurate genome assemblies using only error-prone nanopore data. Purge_dups v1.0.1 (Guan et al. 2020), which uses long-read alignment read-depth and self-alignment to identify assembly artifacts, was used to remove partially duplicated and low-coverage contigs, likely representing errors, to generate a final set of contigs.

### Scaffolding

The initial contig assembly was not entirely chromosome-scale, so Hi-C data from the hybrid were used to scaffold the polished contigs and to identify potential misassembled contigs. The shorter read lengths of Illumina sequence data limited the ability to assign Hi-C reads to either parental haplotype efficiently using unique parental *k*-mers, as for the longer nanopore reads used in the contiging step. Therefore, we instead excluded Hi-C reads from the scaffolding process that contained unique Simmental dam *k*-mers, thereby removing all reads that could have definitively originated from the maternal haplotype and retaining only those that could plausibly have come from the paternal bison haplotype (see Supplementary File S1 for commands).

A scaffolded assembly was generated by mapping the maternal cattle haplotype-excluded Hi-C reads from the hybrid to the polished bison haplotype contigs using bwa v0.7.17 (Li and Durbin 2009) following the Arima mapping pipeline, which maps the ends of each paired read separately and trims chimeric reads (across ligation junctions) based on mapping orientation (https://github.com/ArimaGenomics/mapping_pipeline). The alignments were used to scaffold the assembly with Salsa v2.2 (Ghurye et al. 2017). The Hi-C data were remapped to our scaffolded assembly and PretextMap v0.1 (https://github.com/wtsi-hpag/PretextMap) and PretextView v0.01 (https://github.com/wtsi-hpag/PretextView) were used to generate and visualize the Hi-C matrix and inspect the contiging and scaffolding results.

### Manual Curation and Polishing

We used minimap2 v2.16 (Li 2018) with the parameter -x asm5 to align the scaffolded bison assembly to the latest cattle reference genome, ARS-UCD1.2, with the Y chromosome appended from bosTau5.0.1 (ARS-UCD1.2_Btau5.0.1Y), visualizing the alignment using D-Genies (Cabanettes and Klopp 2018). Cattle have a conserved karyotype with bison (Basrur and Moon 1967), so this alignment, in addition to a Hi-C contact matrix and long- and short-read mapping to the scaffolded assembly, allowed us to identify and manually correct structural errors generated in the scaffolding process. Manual corrections were made by breaking the assembly at existing scaffolding gaps, except for a within-contig break in the case of 1 contiging error, and then properly joining and orienting contigs as supported by the alignment and sequence data with the program CombineFasta (https://github.com/njdbickhart/CombineFasta). We also used the ARS-UCD1.2 alignment to name and orient chromosomes from our scaffolded assembly.

Three rounds of polishing were conducted after the manual curation process, first with nanopore reads using Nanopolish and then 2 rounds of polishing with short-read data using freebayes (Garrison and Marth 2012). Variants called for polishing with both methods were screened with Merfin (https://github.com/arangrhie/merfin) which predicts the *k*-mer consequences of variant calls and validates supported variants. Only *k*-mers from the bison sire haplotype inherited in the hybrid were included for consideration. Filtering out *k*-mers except for those inherited from the sire haplotype in the short-read data using Merfin allowed us to combine the Illumina data from the sire and F_1_ hybrid for polishing, increasing coverage at homozygous sites considered by freebayes without risking haplotype conversion. We then derived a polished consensus by applying homozygous ALT and heterozygous non-REF variants that passed quality filtering [‘QUAL>1 && (GT=“AA” || GT=“Aa”)’] using bcftools (Li et al. 2009), selecting the longest variant at heterozygous non-REF sites.

### Assembly Evaluation

The quality of the genome assembly was assessed in several ways. The completeness of the genome was evaluated using BUSCO v4 (mammalia_odb10; 9226 genes; Simão et al. 2015), which identifies the presence of single-copy orthologs in the assembly. We assessed the base-level error, *k*-mer completeness, and phasing accuracy of our assembly using Merqury v1.0 (Rhie, Walenz et al. 2020). Merqury uses the *k*-mer spectra generated from short-read sequencing data from the individual used in genome sequencing, and parents in the case of trios, to assess the error rate and completeness of the assembly, as *k*-mers found only in the assembly can be assumed to be errors, while *k*-mers found in the sequencing data but not in the assembly represent sequence missing from the assembly. We also used minimap2 to align the assembly to Bison_UMD1.0 to assess sequence similarity between our assembly and the current bison reference. Short-read mapping statistics and variant calls were also used to estimate the quality of the genome, using the bison sire Illumina data mapped against the polished assembly. We used freebayes (Garrison and Marth 2012) and Lumpy-sv 0.3.0 (Layer et al. 2014) to obtain variant calls and FRC_align 1.0.0 (Vezzi et al. 2012) to generate mapping statistics and create feature response curves, as in Bickhart et al. 2017. Assembly evaluation statistics were generated using a collection of custom python and R scripts (https://github.com/njdbickhart/Themis-ASM).

### Annotation

The high levels of sequence conservation between cattle and bison allowed us to lift over the cattle genome annotation to our newly assembled bison genome using Liftoff v1.5.1 (Shumate and Salzberg 2020) to obtain a preliminary annotation of the ARS-UCSC_bison1.0 assembly, before final annotation with the NCBI Eukaryotic Genome Annotation Pipeline. We used the ARS-UCD1.2 annotation (Rosen et al. 2020), removing genes on the X chromosome but adding Y chromosome genes from the Btau_5.0.1 assembly. We applied the parameters -chroms to perform liftover chromosome-by-chromosome and -copies with -sc 0.95 to identify extra gene copies appearing in the assembly, with all other parameters set to the default.

### Identification of Polymorphism and Structural Variation

Sorting reads into parental haplotype prior to assembly makes the process of genome assembly more straightforward, and has the advantage that the resulting genome is phased. Genome phasing has been shown to increase sensitivity of structural variant detection (Chaisson et al. 2019; De Coster and Van Broeckhoven 2019). Trio binning produces 2 fully phased genomes, so there is the additional possibility of identifying structural variation between alleles within the same organism (Low et al. 2020).

Polymorphisms and larger structural variants between bison and other bovids were assessed by using minimap2 to make alignments in a pairwise fashion between all combinations of the bison genome assembly and 5 different bovid genomes, including 3 *Bos taurus taurus*, ARS-UCD1.2 (Rosen et al. 2020), UOA_Angus_1 (Low et al. 2020), and ARS_Simm1.0 (Heaton et al. 2021); 1 *Bos taurus indicus*, UOA_Brahman_1 (Low et al. 2020); and 1 yak (Rice et al. 2020). Variants from this genome–genome alignment were identified using the call function from the minimap2 module paftools.js.

## Results

### Sequencing

The PromethION produced a total of 424.8 Gb of basecalled sequence (487.5 Gb estimated by the PromethION software) across 14.2 M reads generated from F_1_ hybrid fetal DNA, with a mean length of 23.7 Kb (26.55 Gb/flow cell average; average read N50 estimated at 47 kb). Ultra-long template was sequenced with 22 MinION flow cells using the GridION x5, producing 3.7 Gb of reads >100 Kb and 31.1 Gb overall. Total read coverage was estimated at 193.8× (assuming 2.7 Gb genome size).

Short-read data for estimating heterozygosity, polishing, and assembly validation included 346 M Illumina reads (52 Gb; ~19× coverage) generated from the same F_1_ hybrid DNA. Short-read data for sorting the reads into parental bins, and for use in polishing the sire haplotype, included approximately 675 M Illumina reads generated from the sire semen DNA (102 Gb; ~38× coverage) and 980 M reads from the Simmental cow that provided the oocytes (147 Gb; ~54× coverage).

The Hi-C library used for scaffolding the contigs produced ~198 M reads, with a duplication rate of 8%. Approximately 70% of these were high quality, with 11% of read pairs mapping >10 kb apart and 7% mapping to separate contigs. About 22% of pairs had a zero map distance.

### Heterozygosity Estimation

We used GenomeScope to fit the *k-*mer count histograms for each of the parents and the F_1_ hybrid fetus in order to estimate heterozygosity. This yielded heterozygosity estimates of 0.45% for the bison sire, 0.44% for the Simmental dam, and 1.46% for the hybrid (Supplementary Figure S1). This latter level of heterozygosity is comparable to a previous bovid interspecies trio binning assembly (Rice et al. 2020), and higher than prior intraspecific trio binning assemblies (Koren et al. 2018).

### Assembly

A total of 15 084 390 nanopore reads from the F_1_ hybrid were sorted into their respective parental haplotype bins. Remarkably, 99.99% of reads could be successfully assigned to either parental haplotypes, yielding 7 193 718 reads for the bison sire haplotype totaling 216.79 Gb, resulting in ~80× estimated coverage going into the assembly. After the read error correction and trimming steps, 103.35 Gb (approximately 38× coverage) in 1 689 432 reads remained for contiging (Supplementary Figure S2).

**Figure 2.**
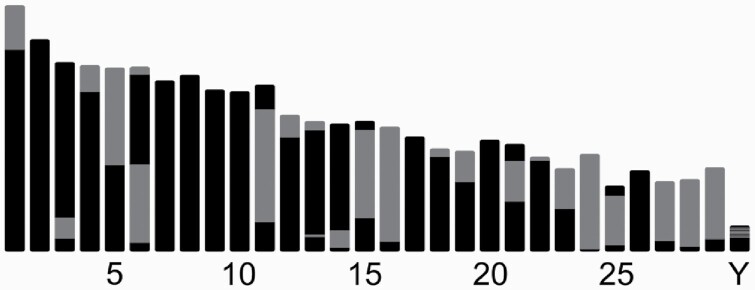
Ideogram of bison genome assembly karyotype, showing placement of contigs within chromosomes as alternating colors (such that color alternates at gaps). Chromosomes shown entirely in black represent those contained within single contigs.

The initial contig assembly produced using Canu resulted in 923 contigs with a total length of 2.65 Gb, with maximum contig length of 135.36 Mb and N50 of 72.88 Mb. The male haplotype bison genome has 30 chromosomes (29 autosomes and the Y chromosome), of which 8 chromosomes were spanned by single ungapped contigs (Figure 2). Nanopolish was used to polish the scaffolds, and purge_dups was then used to remove 100 low-coverage contigs and 35 partially duplicated portions of contigs to create a final set of 823 contigs with an N50 of 73.2 Mb and L50 of 12.

### Scaffolding

The initial assembly was highly contiguous, but some chromosomes were represented by multiple contigs, requiring scaffolding to achieve a full chromosome-scale assembly. Scaffolding used Hi-C data that excluded reads which could be determined to originate from the maternal cattle haplotype, resulting in 124 523 590 unique reads mapping to the contigs, representing approximately 6× coverage of Hi-C data for use in scaffolding with Salsa v2.2. Scaffolding identified 12 positions in contigs that were incorrectly assembled and joined 47 contigs, resulting in an assembly of 788 scaffolds with a scaffold N50 of 83.7 Mb.

The scaffolds were aligned to ARS-UCD1.2_Btau5.0.1Y for manual inspection of the scaffolding process and, in conjunction with the Hi-C contact map (Supplementary File S2) and short- and long-read mapping data, identify and correct errors. All together, we identified and broke 1 contiging error and 4 misjoins and made 19 manual joins, 10 of which were on the Y chromosome (Supplementary File S3). The final assembly, ARS-UCSC_bison1.0 had a total length of 2.65 Gb contained in 775 scaffolds, with a scaffold N50 of 87.8 Mb, L50 of 11, and 49 gaps on the chromosomes, of which 12 were on the Y chromosome (64 gaps total, see Table 2, Figure 2). For comparison, the current bison reference, Bison_UMD1.0, has a length of 2.83 Gb over 128 431 unanchored scaffolds (470 415 contigs), with a scaffold N50 of 7.20 Mb, L50 of 124, and contig N50 of 20 Kb. Note that Bison_UMD1.0 has both X and Y chromosomes, accounting for most of the difference in total length.

**Table 2.** Assembly statistics for final assembly, ARS-UCSC_bison1.0, and current bison reference, Bison_UMD1.0

		ARS-UCSC_bison1.0	Bison_UMD1.0
Genome size (Gb)		2.65	2.83
Contig number		839	470 415
Scaffold number		775	128 431
Contig N50		68.5 Mb	20.0 Kb
Scaffold N50		87.8 Mb	6.87 Mb
Scaffold L50		11	124
Gaps (in chromosomes)		64 (49)	341,984 (NA)
Largest contig		136.1 Mb	203.8 Kb
BUSCO (%)	Complete	88.6	85.6
	Duplicated	1.0	0.9
	Fragmented	2.4	3.9
	Missing	8.0	9.6
*K*-mer statistics	*K*-mer-based QV	38.88	32.21
	*K*-mer completeness	91.35%	93.0%

### Quality Control

Assembly quality was estimated in several ways. First, we used BUSCO to estimate the proportion of genes in the mammalia_od10 BUSCO database present in the assembly. The initial set of contigs produced by Canu had a BUSCO score of 50.4% (50.0% single-copy, 0.4% duplicated). ARS-UCSC_bison1.0 had 89.6% (88.6% S, 1.0% D) of predicted single-copy genes present while Bison_UMD1.0 had a BUSCO of 86.5% (85.6% S, 0.9% D). While BUSCO has some value as a comparative metric between different assemblies, the BUSCO score is quite sensitive to the database used, software version, and mapping parameters, as suggested by Heaton et al. (2021).

A second estimate of assembly quality was performed by a strategy that identifies *k*-mers in the short-read data from the sire and assesses the accuracy and completeness based on *k*-mer content in the final assembly (Supplementary Figure S3; Rhie, Walenz et al. 2020). The *k*-mer-based QV score, a Phred-scaled estimate of base-level error, for ARS-UCSC_bison1.0, was 38.88 (i.e., an estimated error rate of 0.00013), whereas for Bison_UMD1.0 it was 32.21 (estimated error rate of 0.0006). The *k*-mer completeness of ARS-UCSC_bison1.0 was estimated to be 91.35% and that of Bison_UMD1.0 was 93.0%. Some caution should be taken in interpreting these metrics, as both are sensitive to the read set used to construct the *k*-mer spectrum for assembly evaluation. For example, the reads we used here were from the sire of the individual from which ARS-UCSC_bison1.0 was assembled and so our assembly could be expected to only contain *k*-mers that occur in this read set (if no errors were present), whereas Bison_UMD1.0 will contain correct *k*-mers that do not appear in the reads. Additionally, some of these reads originated from the X chromosome, which ARS-UCSC_bison1.0 lacks, perhaps accounting for its reduced completeness estimate.

We also used a *k*-mer-based approach to compare phasing accuracy of the 2 assemblies generated from the F_1_ hybrid by evaluating the presence of unique parental *k*-mers in each assembly (Supplementary Figure S4). The parent-specific *k*-mers found in the final contigs illustrate that the separation of parental haplotypes was successful and that the phasing is correct across all contigs in both the bison and cattle assemblies, by virtue of the lack of dam-specific *k*-mers found in the bison contigs (and lack of sire-specific *k*-mers in the cattle contigs). About 99.3% of the unique parental *k*-mers found in the bison contigs were from the bison haplotype, with the remainder likely consisting largely of base errors. The Simmental assembly had a similar level (99.5%) of phasing accuracy. Finally, we evaluated the assembly with mapping-based approaches. The assembly showed generally high sequence similarity with Bison_UMD1.0 (Supplementary Figure S5) and had fewer errors (Supplementary Table S2; Supplementary Figure S6), while Bison_UMD1.0 had a greater amount of unique sequence (Supplementary Table S2; Supplementary Figure S7).

### Annotation

We used Liftoff to apply the ARS-UCD1.2 assembly annotation with the X chromosome removed but Y chromosome added to our bison assembly, in order to obtain a preliminary annotation of the assembly (Supplementary File S4). Of the 20 402 protein-coding genes in the ARS-UCD1.2, with the X chromosome removed and Btau5.0.1_Y annotation added, 19 890 were successfully lifted over to ARS-UCSC_bison1.0. Two hundred and twelve of these genes had extra copies in ARS-UCSC_bison1.0 not present in the cattle reference annotation.

### Identification of Polymorphism and Structural Variation

We identified variants between our bison genome and other bovid genomes using the minimap2 module paftools.js, which calls variants based on areas within genome–genome alignments where the reference is covered by a single query contig and the alignments meet a minimum size criteria (~85% of the genome in each comparison met this criteria).

A similar number of variants were identified between bison and each of the 4 cattle (representing 4 breeds: Hereford, Angus, Simmental, and Brahman) used (Supplementary Table S3), with ~22 million substitutions and ~2.7 million total insertions/deletions detected between each. There was a slight bias for large (≥1000 bp) insertions versus deletions in bison relative to cattle, indicating extra sequence in the bison genome (~54% of ~9000 indels of this size were insertions). This discrepancy was also observed in each yak–cattle comparison, and bison–yak had a similar number of large insertions as they did deletions, suggesting that there were either sequence expansions in the lineage leading to bison and yak, or deletions on the cattle lineage before the separation taurine and indicine cattle.

Conversely, there was a bias of similar magnitude toward deletions in small indels (1–50 bp) as compared to insertions. This bias was reduced in the bison–Simmental variants relative to the variants called between bison and the other cattle breeds and yak, suggesting that this may be reflective of systematic assembly errors, as the bison and Simmental assemblies were built using the reads from the same F_1_ hybrid with similar assembly approaches.

There were slightly fewer variants detected between bison and yak, as would be expected given their more recent divergence (Wu et al. 2018), with about 19 million substitutions and ~2.5 million indels. For comparison, the taurine cattle had the fewest variants, with ~4.4 million substitutions and ~800 000 indels, of which about 1000 each of insertions and deletions were over 1 Kb. Yak had a similar number of variants with cattle as compared to bison.

## Discussion

The assembly presented here, ARS-UCSC_bison1.0, represents a marked improvement over the current bison reference genome and therefore has the potential to be a valuable resource for evolutionary, ecological, and conservation genetics studies. Bison have been well-studied among nonmodel organisms using genetic information (Ward et al. 2001; Shapiro et al. 2004; Cronin and Leesburg 2016), but such studies have been limited by the absence of a suitable reference genome to uniparental markers or to a handful of nuclear loci. With a high-quality reference genome, similar studies will now be able to fully take advantage of genome-wide information, providing new insight into bison demography and population history (Froese et al. 2017), as well as Beringian biogeography (Heintzman et al. 2016) and paleoecology (Davies et al. 2019) and the response generally of megafauna to climatic shifts (Lorenzen et al. 2011).

ARS-UCSC_bison1.0 is constructed from long reads and is haplotype-resolved. Both of these features should provide greater sensitivity in identifying structural variants (Chaisson et al. 2019) and areas with high diversity or complex allelic variation (Jain et al. 2018; Rice et al. 2020). The potential for achieving 2 separate haplotype-aware assemblies from a single individual allows for the identification of allele-specific structural variation and expression differences (Low et al. 2020). Given that the individual sequenced here is an F_1_ interspecies hybrid, such differences can potentially be used to understand the dynamics of hybrid incompatibility and the maintenance of species boundaries (Schumer et al. 2014; McGirr and Martin 2019).

We anticipate that a high-quality bison reference genome will also assist future management of bison populations in North America. For example, the vast majority of bison exist in commercial herds. A reference genome is necessary for identifying the functional genetic basis behind commercially relevant traits, such as growth rate, for more effective genomic selection (Bickhart et al. 2017). The remainder of the bison population is found on conservation herds on federal and private lands. While these conservation herds represent only a small fraction of the total population (~30 000 bison), such herds encompass the scope of genetic diversity present within the species. These herds are typically small, numbering in the tens or hundreds of individuals, and have historically been managed separately. Due to increased drift in small populations, concerns have arisen that the overall level of diversity in the bison metapopulation is decreasing, and so translocations between conservation herds are being considered (Hartway et al. 2020). As a reference genome allows identification of regions of depleted diversity within the genome, such as runs of homozygosity (Ceballos et al. 2018), or locations of cattle introgression (Corbett-Detig and Nielsen 2017), the genome will allow more informed translocations that best preserve overall genetic diversity (Hartway et al. 2020).

The assembly presented here shows the feasibility of trio binning for generating extremely high-quality reference genomes from nonmodel organisms. As bison are of interest in a range of diverse fields, this assembly provides a new resource that is broadly applicable to a wide array of disciplines.

## Supplementary Material

esab003_suppl_Supplementary_Materials

esab003_suppl_Supplementary-File-S1

esab003_suppl_Supplementary-File-S2

esab003_suppl_Supplementary-File-S3

esab003_suppl_Supplementary-File-S4

## Data Availability

Data generated in this study are available in the NCBI BioProject repository under accessions PRJNA677946 (bison) and PRJNA677947 (Simmental). Fastq files for Simmental cow BHR Lady Sieg C235E (SAMN16825967) and Yellowstone bison bull 709 (SAMN16823422) are deposited in the NCBI Short Read Archive under SRX9528670 and SRX9528561 accessions, respectively.
