# Supplementary material for: A Reference Genome Assembly of American Bison, Bison bison bison
Source: J Hered. 2021 Feb 17;112(2):174–83. doi: 10.1093/jhered/esab003 (PMC8006816; doi:10.1093/jhered/esab003)
Supplement: esab003_suppl_Supplementary_Materials [file esab003_suppl_supplementary_materials.docx]

**Supplementary Material**

**Supplementary Table S1**. Summary of trio sequencing data used in assembly.

**Supplementary Table S2.** Detailed assembly evaluation statistics comparing ARS-UCSC_bison1.0 and Bison_UMD1.0.

**Supplementary Table S3.** Variants called between ARS-UCSC_bison1.0 and other bovines.

**Supplementary Figure S1.** Genomescope results for *k*-mer spectra of bison sire, Simmental dam and F1 hybrid.

**Supplementary Figure S2.** Read length histograms for nanopore reads used in assembly.

**Supplementary Figure S3.** *K*-mer spectra-asm plot for ARS-UCSC_bison1.0.

**Supplementary Figure S4.** Presence of unique parental *k*-mers within bison and Simmental assembly scaffolds.

**Supplementary Figure S5.** Alignment of ARS-UCSC_bison1.0 and Bison_UMD1.0.

**Supplementary Figure S6.** Feature response curve comparing initial bison contigs, ARS-UCSC_bison1.0, and Bison_UMD1.0.

**Supplementary Figure S7.** Variants called between ARS-UCSC_bison1.0, and Bison_UMD1.0.

**Supplementary File S1.** Commands used for sorting reads.

**Supplementary File S2.** Hi-C contact matrix of initial bison contigs.

**Supplementary File S3.** Manual corrections made to scaffolded assembly.

**Supplementary File S4.** Preliminary annotation of ARS-UCSC_bison1.0 lifted over from the cattle reference.

**Supplementary Table S1.** Sequence data used throughout the assembly process.

| **Organism** | **Type of reads** | | **Number of reads** | **Estimated coverage** |
| --- | --- | --- | --- | --- |
| Bison sire | Illumina (2x150) | Shotgun | 675,539,980 | 38 |
| Simmental dam |  | Shotgun | 803,533,237 | 54 |
| F1 hybrid fetus |  | Shotgun | 346,007,778 | 19 |
|  |  | Hi-C (sorted, deduplicated) | 124,523,384 | 6 |
|  | Oxford Nanopore | All | 15,084,390 | 193.8 |
|  |  | Bison haplotype sorted | 7,193,718 | 80 |

**Supplementary Table S2.** Detailed assembly evaluation statistics comparing ARS-UCSC_bison1.0 and Bison_UMD1.0.

| **Q Scores/Features** | **ARS-UCSC_bison1.0** | **Bison_UMD1.0** | **Description** |
| --- | --- | --- | --- |
| CtgNum | 775 | 128431 | Number of contigs |
| TotBases | 2651.61 | 2828.03 | Assembly length in Mbp |
| ContigN50 | 87753233 | 7192658 | Half the length of asm is in ctgs of this size |
| merQV | 38.8765 | 32.2066 | kmer-based Quality |
| merErrorRate | 0.000129524 | 0.000601648 | kmer-based error rate |
| merCompleteness | 91.3475 | 92.9958 | Proportion of complete assembly based on kmers |
| baseQV | 34.32 | 29.22 | SNP and INDEL Quality value |
| unmap% | 1.85 | 0.85 | Percentage of short-reads unmapped |
| COMPLETESC | 88.6 | 85.6 | Percent of complete, single-copy BUSCOs |
| COMPLETEDUP | 1 | 0.9 | Percent of complete, duplicated BUSCOs |
| FRAGMENT | 2.4 | 3.9 | Percent of fragmented BUSCOs |
| MISSING | 8 | 9.6 | Percent of missing BUSCOs |
| LOW_COV_PE | 41458 | 195754 | Low read COV areas |
| LOW_NORM_COV_PE | 41803 | 223789 | Low COV of normal PE reads |
| HIGH_SPAN_PE | 4062 | 114688 | Regions with high numbers of inter-contig PE reads |
| HIGH_COV_PE | 13454 | 40351 | Regions with High read coverage |
| HIGH_NORM_COV_PE | 12449 | 32418 | Regions with high coverage of normal PE reads |
| HIGH_OUTIE_PE | 82 | 4107 | Regions with high counts of improperly paired reads |
| HIGH_SINGLE_PE | 53 | 4403 | Regions with high counts of single unmapped reads |
| STRECH_PE | 19611 | 127623 | Regions with high Comp/Expansion (CE) statistics |
| COMPR_PE | 19926 | 19160 | Regions with low Comp/Expansion (CE) statistics |
| SVDEL | 2886 | 28713 | Number of deletion SVs |
| SVDUP | 737 | 19472 | Number of Duplication SVs |
| SVBND | 6592 | 192206 | Number of Complex SVs |

**Supplementary Table S3.** Variants called using minimap2 between ARS-UCSC_bison1.0 and genome assemblies from five bovines, four different cattle breeds and one yak. Variants are stratified by class (substitution, insertion, deletion) and size.

|  |  | **Reference** | | | | | **Variant class** |  |
| --- | --- | --- | --- | --- | --- | --- | --- | --- |
|  |  | Hereford | Angus | Yak | Simmental | Brahman |  | **Size** |
| **Query** | Bison | 22.04 M | 21.96 M | 18.68 M | 21.74 M | 22.01 M | **substitution** | **-** |
|  |  | 738,917 | 715,156 | 668,054 | 657,372 | 718,768 | **deletion** | **1bp** |
|  |  | 576,140 | 585,235 | 590,288 | 642,241 | 577,626 | **insertion** |  |
|  |  | 205,197 | 201,896 | 180,361 | 198,542 | 202,684 | **deletion** | **2bp** |
|  |  | 189,223 | 189,798 | 181,031 | 186,970 | 191,414 | **insertion** |  |
|  |  | 486,848 | 487,580 | 416,965 | 464,572 | 489,347 | **deletion** | **[3,50)** |
|  |  | 434,420 | 434,587 | 385,222 | 432,557 | 435,997 | **insertion** |  |
|  |  | 23,685 | 23,743 | 20,782 | 23,768 | 23,798 | **deletion** | **[50,1000)** |
|  |  | 22,720 | 22,388 | 18,844 | 22,919 | 22,338 | **insertion** |  |
|  |  | 4,129 | 4,127 | 4,299 | 4,164 | 4,111 | **deletion** | **>=1000** |
|  |  | 4,931 | 4,958 | 4,255 | 4,976 | 4,915 | **insertion** |  |


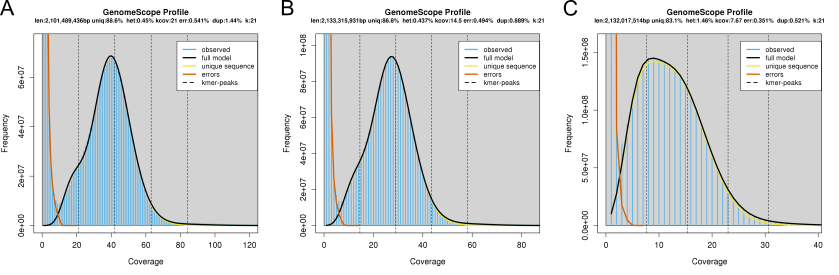


**Supplementary Figure S1**. Genomescope results of fitting the *k*-mer histogram for the bison sire (A), Simmental dam (B), and F1 hybrid (C).


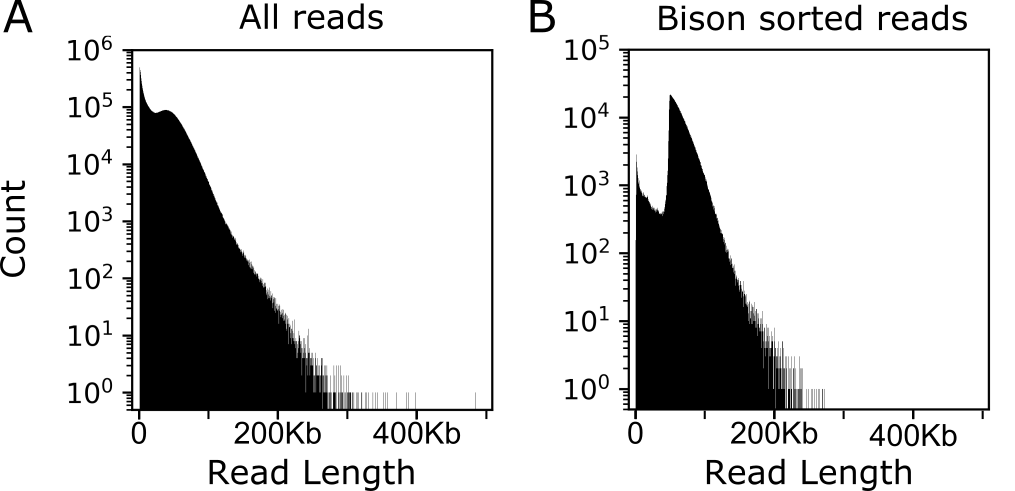


**Supplementary Figure S2**. Read length histograms of nanopore reads generated from the F1 hybrid. A) shows all nanopore reads, while B) depicts only bison haplotype-sorted, Canu error-corrected reads used for unitigging. Note the log scale on the y-axis.

**
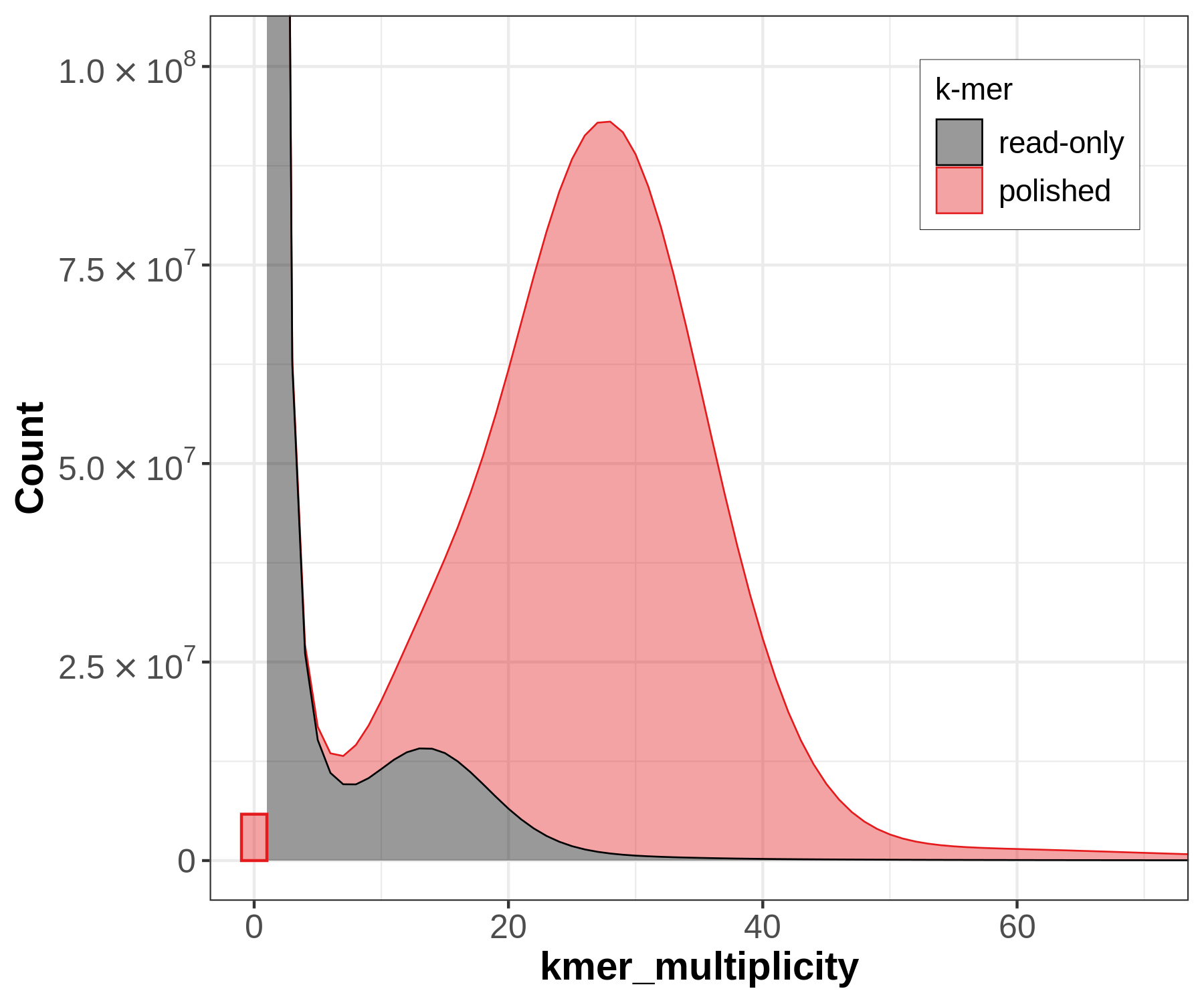
**

**Supplementary Figure S3.** *K*-mer spectra-asm plot for the ARS-UCSC_bison1.0 assembly, showing the *k*-mers present in the read set (derived from short reads from the bison sire). *K*-mers present in the assembly are shown in red and those present only in the reads are shown in gray. *K*-mers seen only in the assembly are those with multiplicity of 0.


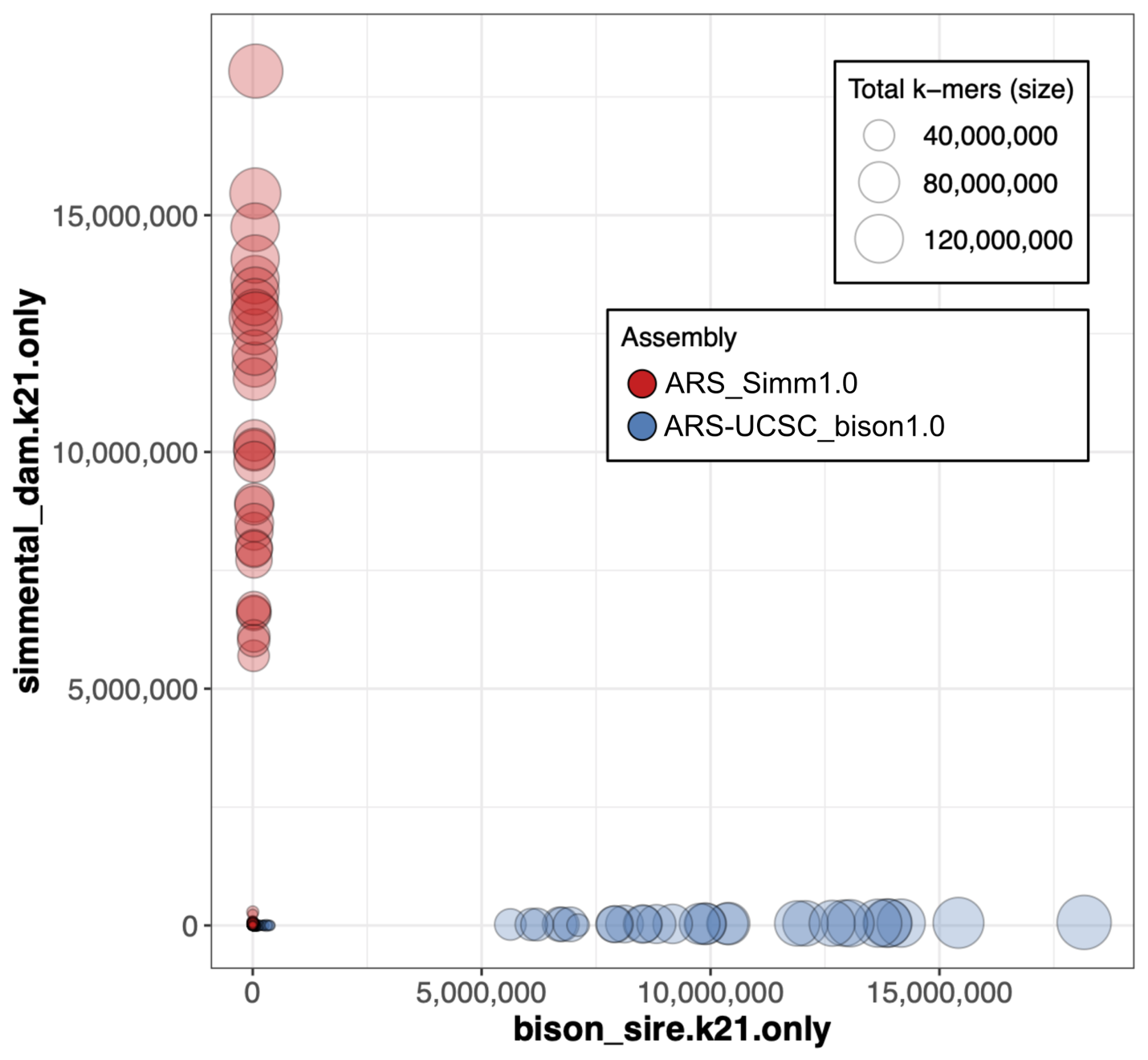
**Supplementary Figure S4.** Depiction of all contigs of the two haplotypes, bison and Simmental (Heaton *et al*, *submitted*), assembled from the F1 hybrid, by the number of unique haplotypic *k*-mers found in the contig. Each circle represents a contig and is colored by assembly of origin, plotted by the number of bison haplotype *k*-mers on the x-axis value and Simmental *k*-mers on the y-axis. The size of the circle represents the total number of such *k*-mers found in the contig. Greater alignment with either axis for each respective assembly (bison on the x and Simmental on the y), indicates more effective phasing.


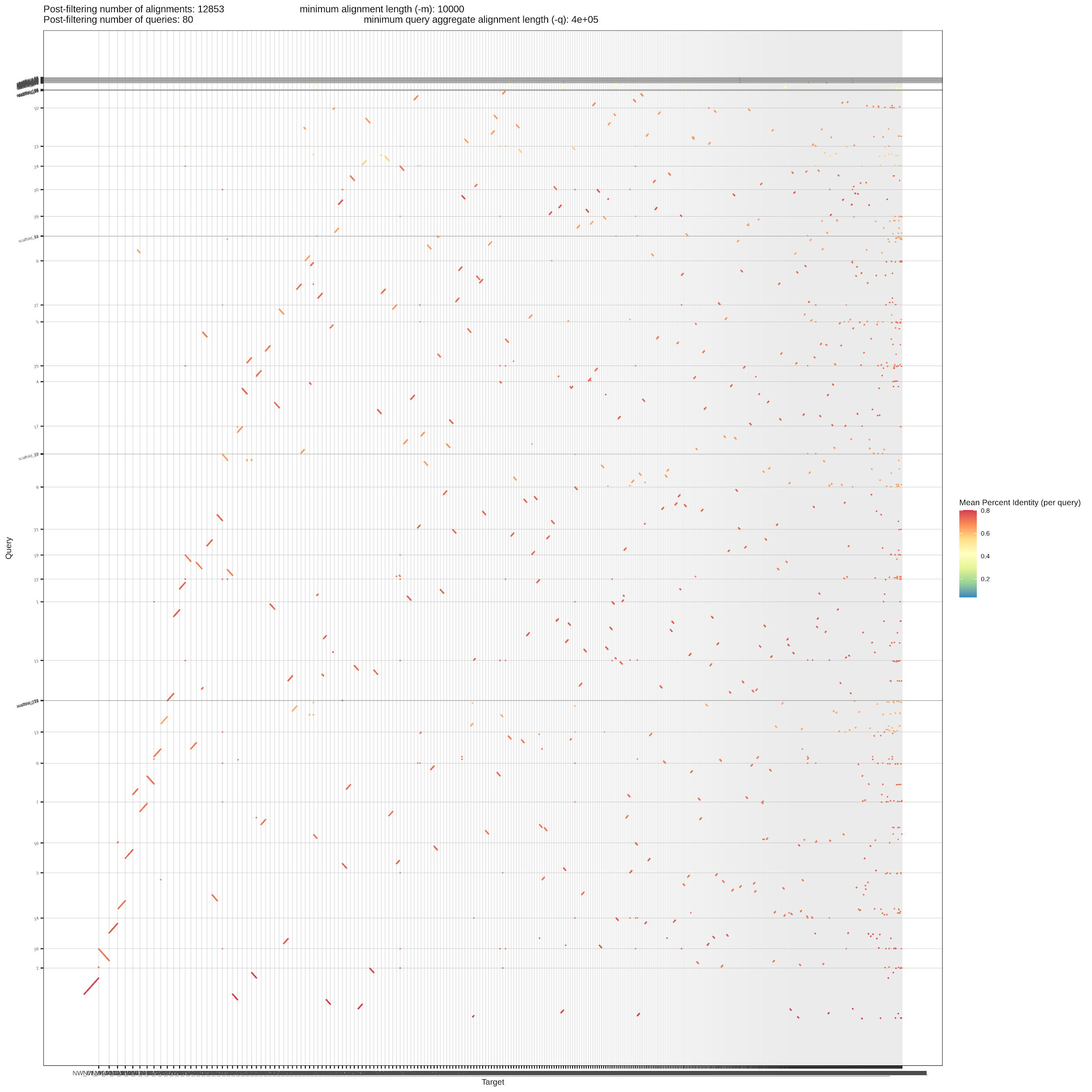


**Supplementary Figure S5.** Dotplot visualizing an alignment of ARS-UCSC_bison1.0 (y-axis) to the Bison_UMD1.0 reference (x-axis), with sequence similarity between aligned segments depicted by color.


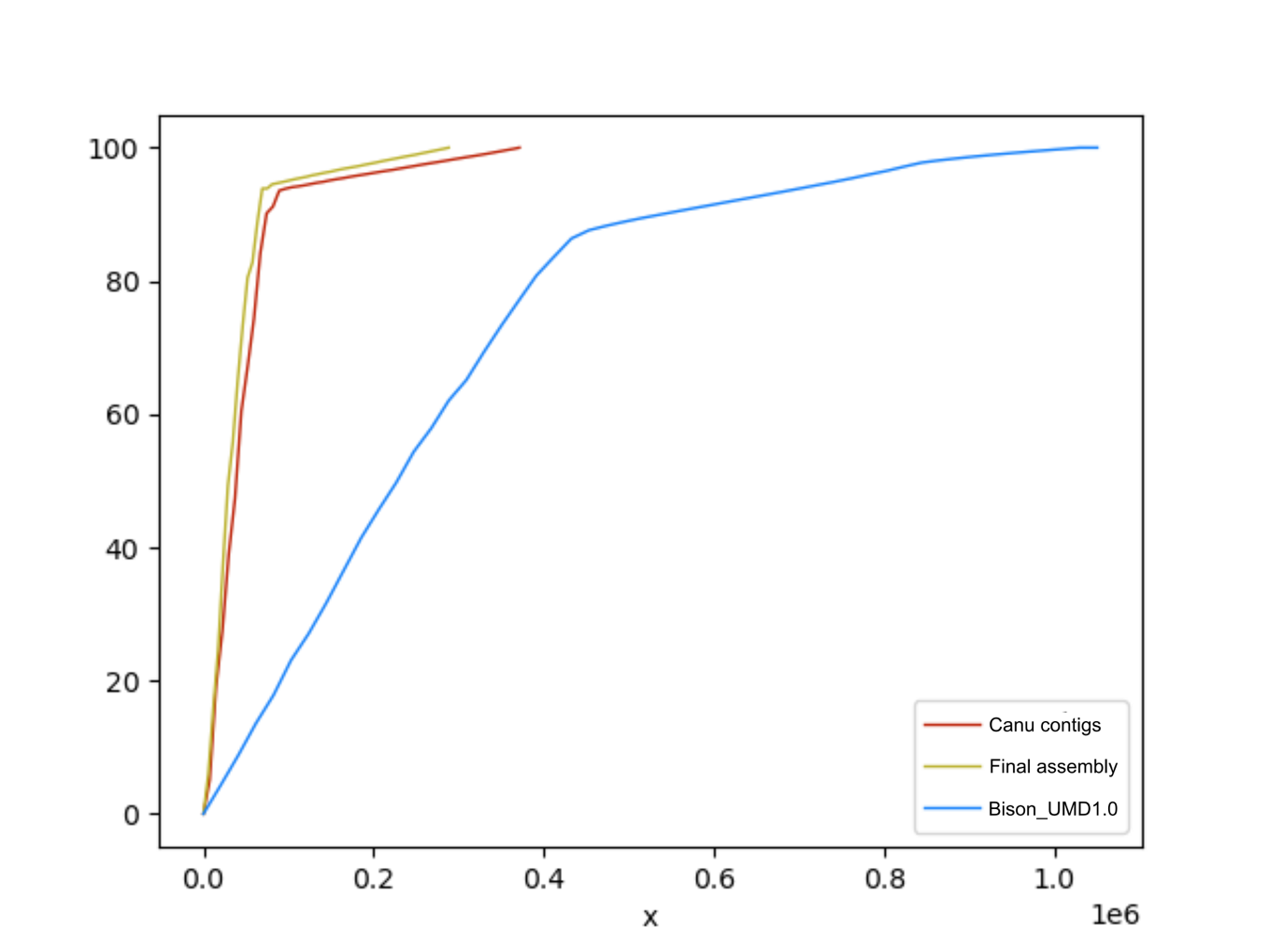


**Supplementary Figure S6.** Feature response curve showing the number of total errors (x-axis) relative to genome length (y-axis) for the initial contig assembly generated by Canu, ARS-UCSC_bison1.0, and Bison_UMD1.0. Errors were identified using FRC_align [(Vezzi *et al.*, 2012)](https://paperpile.com/c/vzcOA1/dKcI). Contigs are sorted by length, so that a curve on the diagonal indicates equal rate of error throughout contigs of all sizes, whereas curves with peaks further up and to the left indicate lower rates of error on larger contigs, indicating higher assembly quality.


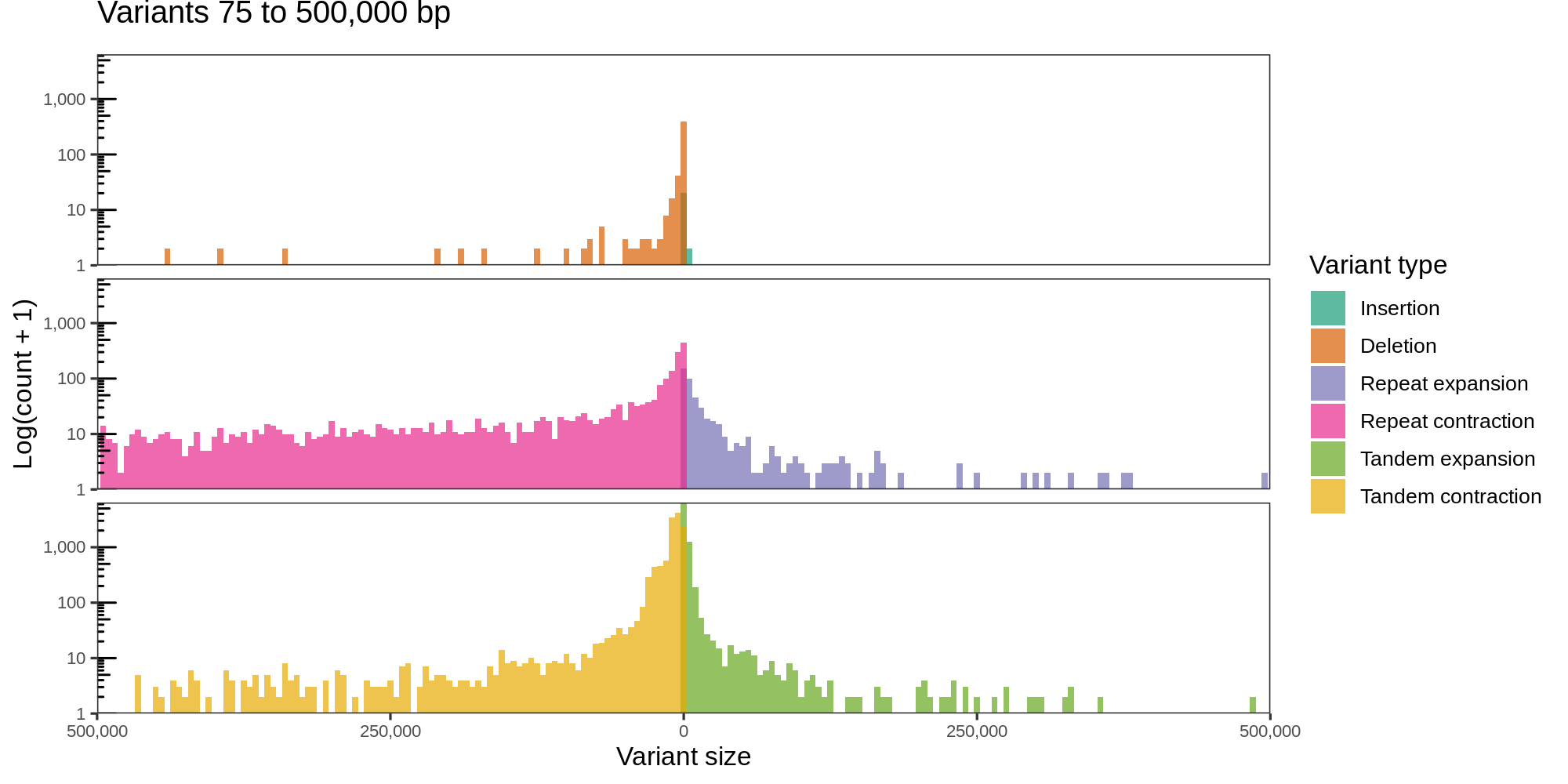


**Supplementary Figure S7.** Variants between ARS-UCSC_bison1.0 and Bison_UMD1.0 called with minimap2, aligning ARS-UCSC_bison1.0 to Bison_UMD1.0. Insertions and expansions represent unique sequence present in ARS-UCSC_bison1.0, while deletions and contractions represent unique sequence in Bison_UMD1.0. Most unique sequence is present in Bison_UMD1.0.

**Supplementary File S1.** Commands used for read binning to the bison haplotype for the initial Canu assembly and exclusion of Simmental haplotype Hi-C reads for scaffolding.

Filename: S1_binning_commands.pdf

**Supplementary File S2.** Pretext file of Hi-C contact matrix for visualization with PretextView.

Filename: bison_sire_hic_excluded_min6.scaffold_rep1.bam.pretext

**Supplementary File S3.** Bed file used as input to CombineFasta to make manual edits to the assembly following scaffolding.

Filename: sire_salsa_scaffolds_plan.bed

**Supplementary File S4.** GFF from a liftover of the ARS-UCD1.2_Btau5.0.1Y annotation using Liftoff, as a preliminary annotation for ARS-UCSC_bison1.0.

Filename: bison.liftoff.gff.gz
