# Supplementary material for: A Reference Genome Assembly of American Bison, Bison bison bison
Source: J Hered. 2021 Feb 17;112(2):174–83. doi: 10.1093/jhered/esab003 (PMC8006816; doi:10.1093/jhered/esab003)
Supplement: esab003_suppl_Supplementary-File-S1 [file esab003_suppl_supplementary-file-s1.pdf]

Nanopore read binning

canu 'corMhapOptions=--threshold 0.8 --ordered-sketch-size 1000 --ordered-kmer-size 14' saveOverlaps=true correctedErrorRate=0.105 'stageDirectory=$TMPDIR' -p BisonSimmental -d assembly genomesize=2.7g -haplotypeBison_sire bison_sire.fastq -haplotypeSimmental_dam Simmental_dam.fq -nanopore-raw bison_simmental.ul.ont.fasta.gz

Meryl Hi-C read binning

meryl-lookup -exclude -mers simmental_dam.only.meryl -sequence hic_R1.fastq.gz -sequence2 hic_R2.fastq.gz -r2 Bison_sire_hic-dam-excluded.R2.fastq.gz | pigz -c > Bison_sire_hic-dam-excluded.R1.fastq.gz
